# A method to improve dose gradient for robotic radiosurgery

**DOI:** 10.1120/jacmp.v16i6.5748

**Published:** 2015-11-08

**Authors:** Tianfang Li, Cihat Ozhasoglu, Steven Burton, John Flickinger, Dwight E. Heron, M. Saiful Huq

**Affiliations:** ^1^ Department of Radiation Oncology University of Pittsburgh Cancer Institute Pittsburgh PA USA

**Keywords:** CyberKnife, Iris, Paddick gradient index, conformity index

## Abstract

For targets with substantial volume, collimators of relatively large size are usually selected to minimize the treatment time in robotic radiosurgery. Their large penumbrae may adversely affect the dose gradient around the target. In this study, we implement and evaluate an inner‐shell planning method to increase the dose gradient and reduce dose to normal tissues. Ten patients previously treated with CyberKnife M6 system were randomly selected with the only criterion being that PTV be larger than $2\;{\text{cm}}^{3}$. A new plan was generated for each patient in which the PTV was split into two regions: a 5 mm inner shell and a core, and a 7.5 mm Iris collimator was exclusively applied to the shell, with other appropriate collimators applied to the core depending on its size. The optimization objective, functions, and constraints were the same as in the corresponding clinical plan. The results were analyzed for V12 Gy, V9 Gy, V5 Gy, and gradient index (GI). Volume reduction was found for the inner‐shell method at all studied dose levels as compared to the clinical plans. The absolute dose‐volume reduction ranged from $0.05\;{\text{cm}}^{3}$ to $18.5\;{\text{cm}}^{3}$ with a mean of $5.6\;{\text{cm}}^{3}$ for 12 Gy, from $0.2\;{\text{cm}}^{3}$ to $38.1\;{\text{cm}}^{3}$ with a mean of $9.8\;{\text{cm}}^{3}$ for 9 Gy, and from $1.5\;{\text{cm}}^{3}$ to $115.7\;{\text{cm}}^{3}$ with a mean of $24.8\;{\text{cm}}^{3}$ for 5 Gy, respectively. The GI reduction ranged from 3.2% to 23.6%, with a mean of 12.6%. Paired *t*‐test for GI has a p‐value of 0.0014. The range for treatment time increase is from ‐3 min to 20 min, with a mean of 7.0 min. We conclude that irradiating the PTV periphery exclusively with the 7.5 mm Iris collimator, rather than applying mixed collimators to the whole PTV, can substantially improve the dose gradient, while maintaining good coverage, conformity, and reasonable treatment time.

PACS number: 87.55.de

## I. INTRODUCTION

The CyberKnife robotic radiosurgery unit (Accuray Inc., Sunnyvale, CA) is an advanced image‐guided radiation therapy system, which uses near‐real‐time imaging to align the radiation beam with the target continually throughout the treatment (typically every 30–60 s).^(1)^ The nonisocentric, noncoplanar ability of the system also facilitates fast dose falloff near the boundary of the target, a dosimetric property especially important for stereotactic radiotherapy to help minimize the dose to nearby critical structures, and usually can be represented by the gradient index (GI).^2^, ^3^ Paddick and Lippitz^(4)^ pointed out that a more favorable GI, with a steeper dose gradient, could lead to a lower applied radiation dose to the healthy brain and ultimately to a lower complication rate. This is particularly valuable in critical anatomical locations or larger volumes where the GI can be used in selecting the plan with the lowest "penumbra dose." They suggest a GI of less than 3.0 for common GammaKnife surgery (GKS).^(4)^ Similarly, Wagner et al.^(5)^ proposed a mixed score index averaging a conformity score and a gradient score for ranking rival stereotactic radiosurgery plans. Although recent univariate and multivariate analysis has not shown the significance of GI as a predictive parameter for complication in brain metastases treatment^6^, ^7^, ^8^ based on GKS patient studies, it is generally believed a fast dose falloff is critical for stereotactic radiosurgery/radiotherapy in most cases.^9^, ^10^, ^11^

Traditionally, fixed divergent circular collimators (cones) have been employed to shape X‐ray beams used for CyberKnife, which have very low collimator transmission and sharp penumbrae. Although the system allows up to three fixed cones to be combined in a single treatment plan (per target), using multiple fixed collimators with the CyberKnife system is usually time‐consuming, because the treatment is automatically divided into multiple traversals of the robotic manipulator around the patient, and collimators need to be changed manually for each traversal. This problem was greatly alleviated with the introduction of Iris Variable Aperture Collimator (Accuray Inc.), which automatically changes the field size along a single traversal. While the Iris system makes it easier to combine multiple collimators in one plan, the beam selection algorithm does not automatically guarantee that the smallest collimator is applied to the target boundary. Since a larger aperture collimator implies a larger penumbra, which contributes to a slow dose falloff, especially for a large treatment volume, a more focused collimator is preferred to irradiate the target boundary. In this work, we investigate an inner‐shell (i.e., a shell inside the target) method to improve the dose gradient near the target for Iris‐based planning.

## II. MATERIALS AND METHODS

### A. Robotic radiosurgery unit and treatment planning system

The robotic radiosurgery unit used in this investigation is CyberKnife (M6 FIM), which consists of a linear accelerator mounted on a robotic manipulator and equipped with three types of collimators: the fixed‐cone collimator, the Iris variable‐aperture collimator, and the InCise (Accuray Inc.) multileaf collimator. The treatment planning process was carried out with a dedicated treatment planning system (TPS), Multiplan version 5.1.3 (Accuray Inc.), which incorporates three planning methods — isocentric, conformal, and sequential; and three dose calculation methods — ray tracing, Monte Carlo, and finite‐size pencil beam (for MLC‐based plan only). As in the previous version of Multiplan, there are 12 collimator sizes available for the fixed cones or Iris collimator, which are 5, 7.5, 10, 12.5, 15, 20, 25, 30, 35, 40, 50, and 60 mm in diameter measured at a nominal treatment distance of 800 mm. However, due to the concern of the relatively large uncertainty on the output and the geometry reproducibility, the 5 mm Iris collimator must be combined with other Iris collimator sizes in a single plan and its usage is restricted to approximately 10% of the total MU, so that there is no more than a 2% error relative to the prescription dose for the plan.

In this investigation, the sequential method was used for all plans. After the initial setup of beam directions and choice of collimator size, the inverse treatment‐planning algorithm selects beam weights for each direction by minimizing a series of linear cost functions one at a time sequentially, such as maximizing the minimum dose to target (OMI) or maximizing the mean dose, which is termed as "optimize coverage (OCO)," or "optimize homogeneity (OHI)" in the software. The Simplex algorithm is used to solve the linear programming problem.^(12)^ The upper bounding constraints are set prior to the optimization, such as the maximum doses for the target volumes and the organs at risk. Other objectives are set in the sequential optimization steps. Another important constraint is the MU‐per‐beam limit, which specifies the maximum allowed MUs for a single beam direction. That helps to reduce isodose lines showing up as streaks in the direction of beam entry points, and hot spots in the vicinity of the beam entry points just below the skin surface.

### B. Inner‐shell planning

**Figure 1:**
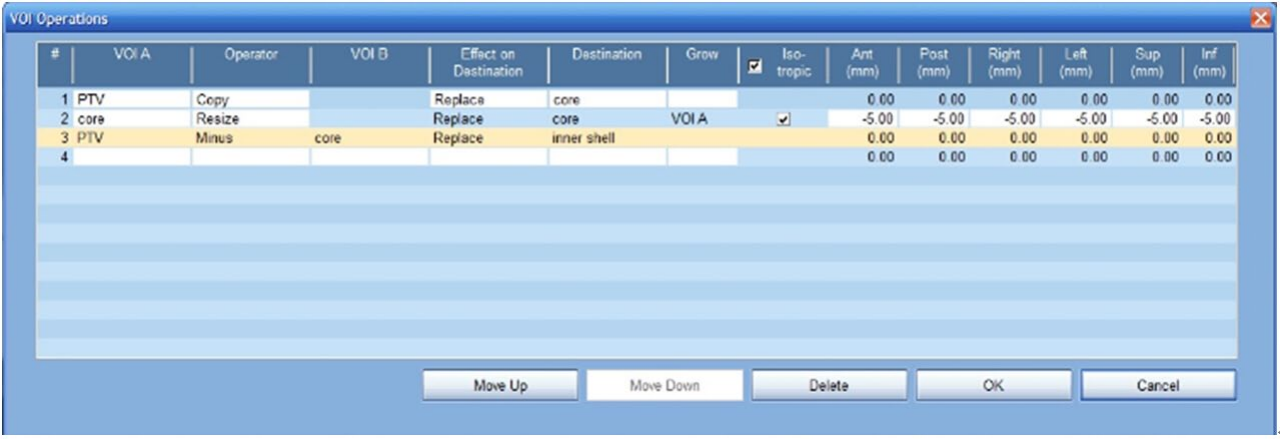
Steps to divide the target into an inner shell and a core using VOI operations with Multiplan 5.1. In this example, the "core" is isotropically reduced by 5 mm in each direction (A/P, R/L, and S/I), and the "inner shell" is then generated by subtraction.

The aim of the inner‐shell approach is to use beams with the smallest size of collimator to irradiate the periphery of the target, so the penumbra effect of the larger collimators is contained within the PTV. To do so, the PTV is divided into two regions: an inner shell and a core. The core is created by shrinking the PTV by 5 mm using 3D erosion, and the shell is then obtained by subtracting the core from the PTV, as indicated by the steps shown in Fig. 1. For all plans, once the inner shell is created a 7.5 mm Iris collimator is exclusively applied to the shell during the collimator selection step, no matter what size the whole PTV is; the selection of the collimator size for the core depends on the size of the core. The beam targeting points for the 7.5 mm Iris collimator are randomly distributed over the shell surface, and the points for other collimators are distributed over a smaller surface of the core inside.

In this investigation, all patient clinical plans were generated with Iris collimator of multiple aperture sizes, the template path set was full path, and the tracking method was 6D skull tracking. The MU per beam was limited to 200∼300, depending the prescription, which was typically less than 10% of the maximum dose in cGy. Constraints were set so that the prescribed dose was 80% of the target maximum, and critical structures if close to the target satisfied the physician's requirements. All plans used in this study had coverage over 98%. In order to control the dose falloff speed for the clinical plans, two to three shells around the target were generated automatically using the TPS provided function. The maximum dose constraints were assigned to these shells, and decreased manually during multiple trials to see if a better plan could be obtained without compromising the coverage significantly. Typically, three to five reoptimizations were tried in this step for finding the final clinical plan with the desired dose falloff. As to the corresponding new plan using the inner‐shell method, it was generated for each patient with the exactly same setup as the clinical plan, except that an additional 7.5 mm Iris collimator was selected aiming only at the inner shell of the target. Thus, the differences between the two plans are solely due to the small collimator enforced on the PTV boundary.

### C. Patient group

Computed tomography (CT) data from 10 patients previously treated for brain tumor with the robotic radiosurgery unit were used. Treatment planning images were based on a CyberKnife brain scan protocol with a slice spacing of 1.25 mm. The selection criterion was that the PTV size needed to be larger than $2\;{\text{cm}}^{3}$, in order to split it into two meaningful parts, as mentioned above. The actual planning target volume PTV ranged from 2.5 to $63.2\;{\text{cm}}^{3}$ (mean $16.2\;{\text{cm}}^{3}$). The GTV for each patient was delineated based on an MRI fusion acquired typically within four days, and the PTV was the same as GTV. A 6D skull tracking method is used, and all treatment plans were constructed to deliver 18 Gy to 24 Gy in 1 or 3 fractions, prescribed to the 80% isodose level with the maximum dose at 100%.

### D. Plan evaluation and comparison

The conformity index (CI), PTV coverage, Paddick GI, V5 Gy, V9 Gy, and V12 Gy were calculated for each plan. The CI was calculated as the ratio between the volume receiving the prescription dose (80%) or more and the volume of the PTV; the GI was calculated as the ratio of the volume of half the prescription isodose to the volume of the prescription isodose.^(3)^ For four patients the maximum, mean, and minimum doses to organs at risk, such as brain stem, chiasm, and skin, were also evaluated; for the other patients this was of no concern. Since dose gradient is usually not constant, depending on whether it is in high‐dose region or low‐dose region, parameters such as the GI, V5 Gy, V9 Gy, and V12 Gy were all included to compare the dose falloff speed properties. These parameters may also be related to brain complications.^2^, ^3^, ^4^ The delivery times per fraction were included in the comparison for practical concern. As mentioned earlier, to evaluate the effectiveness of the inner‐shell method, every step of the clinical plan setup was kept the same, such as the alignment, beam paths, collimator selection (for PTV and the inner core), and constraints, except that one 7.5 mm Iris collimator was added to aim at the periphery shell of the target only.

## III. RESULTS

In Table 1, the volume covered by at least 12 Gy, 9 Gy, and 5 Gy, together with the GIs, are compared for the clinical plans and the inner‐shell plans for all ten patients. From the table, the dose reduction to normal brain tissue can be seen at all dose levels. The absolute dose‐volume reduction for 12 Gy ranged from $0.05\;{\text{cm}}^{3}$ to $18.5\;{\text{cm}}^{3}$, with mean $5.6\;{\text{cm}}^{3}$; the dose‐volume reduction for 9 Gy ranged from $0.2\;{\text{cm}}^{3}$ to $38.1\;{\text{cm}}^{3}$ with a mean of $9.8\;{\text{cm}}^{3}$; the dose‐volume reduction for 5 Gy ranged from $1.5\;{\text{cm}}^{3}$ to $115.7\;{\text{cm}}^{3}$ with a mean of $24.8\;{\text{cm}}^{3}$. The GI reduction ranges from 3.2% to 23.6%, with a mean of 12.6%. Paired *t*‐test for GI has a p‐value of 0.0014, showing the statistical significance of the new method in improving the dose gradient.

**Figure 2:**
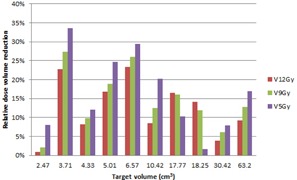
Relationship between dose‐volume reduction and the target volume. Red shows the percentage volume reduction for 12 Gy isodose volume for various target volumes. The same applies to green and blue for 9 Gy and 5 Gy isodose volumes, respectively.

Figure 2 plots the relation between target volume and the relative dose reduction to normal tissue due to the use of the inner‐shell method. The volume reduction is up to 24% for 12 Gy, 26% for 9 Gy, and 34% for 5 Gy regions. However, no obvious correlation can be found between the degree of dose reduction to normal tissue and the size of the target. Generally speaking, the larger the target is, the larger the applied collimators will be in the clinical plan. However, in most cases in this study, two or three collimators (aperture sizes) were used in the clinical plans, including the 7.5 mm collimator. Since the 7.5 mm collimator may already be used in the original clinical plan, and because the collimators' targeting points were randomly distributed on the target, it is difficult to predict how much the improvement will be with the new planning method by looking at the target volume only.

**Table 1:** The target volumes; the volumes covered by at least 12 Gy (V12), 9 Gy (V9), and 5 Gy (V5); and the gradient index (GI) are shown. The lower‐case letters "c" and "i" indicate the clinical plans and the inner‐shell plans, respectively

	$Vol\;(cm^{3})$	$V12(c)\;(cm^{3})$	$V12(i)\;(cm^{3})$	$V9(c)\;(cm^{3})$	$V9(i)\;(cm^{3})$	$V5(c)\;(cm^{3})$	$V5(i)\;(cm^{3})$	GI(c)	GI(i)
1	6.57	17.23	13.21	25.03	18.50	58.22	41.12	3.26	2.49
2	10.42	36.55	33.44	56.18	49.12	146.23	116.67	3.46	3.12
3	2.47	5.48	5.43	7.89	7.72	18.39	16.90	2.91	2.82
4	3.71	11.56	8.93	17.64	12.81	41.32	27.47	3.74	2.90
5	18.25	60.25	51.71	85.96	75.69	199.15	195.81	2.95	2.55
6	4.33	10.05	9.218	14.37	12.96	30.79	27.08	3.02	2.72
7	30.42	90.46	86.87	130.75	122.66	309.84	285.24	2.68	2.55
8	63.2	199.41	180.95	297.52	259.45	680.64	564.92	3.16	2.93
9	17.77	68.26	56.97	101.99	85.53	220.07	197.32	3.31	2.84
10	5.01	18.55	15.42	27.95	22.66	64.62	48.68	2.89	2.39

For six out of the ten patients, the targets were isolated; but the remaining four patients in this study had critical structures nearby, where the maximum dose constraint was assigned to the corresponding structure in each case for beam weights optimization. The impact of the inner‐shell method on the critical structure is demonstrated in Table 2. In general, whenever there is a critical structure near the target, a hard dose constraint is typically applied based on the physician's requirement. The optimization process searches for the optimal solution to the objective function defined in the sequential method while satisfying these constraints. Therefore, the maximum point doses for different methods are typically similar, providing the constraints are set the same way. However, the different beam arrangements still result in dose distribution differences. It is found that, for all four cases, the mean dose decreases consistently, and the maximum reduction to the mean dose is dramatic (up to 58%).

Because more beams with smaller aperture are applied to the target with the inner‐shell method, the treatment time is likely to be longer as compared to the clinical treatment. Table 3 lists the treatment time for the clinical plans and the inner‐shell plans, together with their conformity and gradient indices to demonstrate the benefit and the associated cost. The range for time increase is from ‐3 min to 20 min, with median of 6.0 min. The average treatment time increase is 12.8%.

**Table 2:** The minimum, mean, and maximum dose for the critical structure from the clinical plans (indexed by "c") are compared to the doses computed from the inner‐shell plans (indexed by "i")

*Critical Structure*	*Min (c) (cGy)*	*Min (i) (cGy)*	*Mean (c) (cGy)*	*Mean (i) (cGy)*	*Max (c) (cGy)*	*Max (i) (cGy)*
P#2 (Brainstem)	52.31	19.75	479.21	349.36	1623.19	1619.30
P#3 (Chiasm)	46.05	16.30	173.92	72.79	292.76	208.50
P#5 (Chiasm)	107.53	52	182.30	148.01	317.65	298.92
P#7 (Skin)	55.66	54.43	721.51	684.76	1769.65	1721.31

**Table 3:** The conformity index (CI), coverage, and treatment time from the clinical plans (indexed by "c") are compared to the corresponding parameters derived from the inner‐shell plans (indexed by "i"), respectively. The differences in gradient index (ΔGI) between the clinical and inner‐shell plans are also shown

	*CI (c)*	*CI (i)*	*Coverage (c) (%)*	*Coverage (i) (%)*	ΔGI	*Time (c) (min)*	*Time (i) (min)*
1	1.17	1.13	99.45	99.70	24%	43	59
2	1.23	1.22	98.13	98.00	10%	62	59
3	1.27	1.28	99.49	99.52	3%	56	57
4	1.27	1.19	99.93	99.76	23%	51	71
5	1.12	1.11	99.75	99.68	14%	42	48
6	1.10	1.10	99.90	99.63	10%	52	66
7	1.11	1.12	99.27	99.43	5%	67	72
8	1.20	1.15	99.86	99.65	7%	65	62
9	1.16	1.13	99.66	99.64	14%	53	61
10	1.28	1.29	99.64	99.73	17%	56	62

## IV. DISCUSSION

In this study we have applied and investigated the proposed inner‐shell technique for treatment of brain tumors using CyberKnife. The technique can be applied to other tumor sites as well, both intra‐ and extracranial. For brain treatment, the study has demonstrated consistent dose‐volume reduction from 5 Gy to 12 Gy. For other treatments the technique may also prove important, especially for its ability to spare an organ at risk (OAR); for example, to lower spinal cord dose for spine treatments, or to reduce the skin dose for treatment of tumors at shallow depth.

For lung SBRT, Poll et al.^(13)^ have showed the benefit of using a two‐cone planning method, with the smaller cone targeting the periphery of PTV and the larger cone targeting the inner core, which is very similar to the method described here. The main difference is that our study is based on the Iris collimators instead of the fixed cones. Furthermore, our focus here is to improve the dose falloff speed rather than to save MUs. More importantly, we enforce specifically the 7.5 mm Iris collimator to the target periphery (rather than an arbitrary small collimator as proposed by Poll and colleagues) to increase the dose gradient as much as possible, regardless of the target size. For SBRT of lung tumor or other sites like liver or prostate, where large‐size collimators are frequently used, the inner‐shell method may offer significant improvement in the dose distribution. However, the treatment time increase may be unacceptable in certain cases with extremely large targets. In these situations, the next‐smallest Iris collimator (for example, 10 mm aperture size) may be selected to replace the 7.5 mm Iris collimator.

Note that, for nonisocentric setups, the distance of the source to the target is not constant, which results in the effective size of the collimator at the target plane being different from the nominal value and varied from beam to beam; therefore, in order to apply the 7.5 mm Iris collimator to the inner shell of the target, we recommend in this study using a shell thickness of 5 mm to assure full coverage. However, with the increase of the target size, and especially for tumors in the body where the average source‐to‐target distance is typically larger than 800 mm, shell thicknesses up to 10 mm may be used as well. In our experience, the inner‐shell method is insensitive to the selection of shell thickness.

## V. CONCLUSIONS

To improve the dose gradient and reduce the dose to normal brain tissue in CyberKnife treatments, we have investigated a planning strategy whereby the target was split into a shell and a core, and the shell was irradiated exclusively with the 7.5 mm Iris collimator instead of applying mixed collimators to the whole PTV. For brain cancer patients treated with skull tracking in this study, the GI was reduced on average by 12.6% (range 3.2%‐23.6%), with low dose volume covered by 5 Gy reduced even more (by up to 33.5%), while maintaining good plan quality for the target, and the treatment time was increased 12.8% on average (median of 6 min).
